# Colchicine can keep the viability of bacteria in mastitic milk by preventing leukocyte phagocytosis in dairy cow and goat

**DOI:** 10.3389/fvets.2024.1469586

**Published:** 2024-10-17

**Authors:** Keiichi Hisaeda, Masato Hirano, Naoki Suzuki, Naoki Isobe

**Affiliations:** ^1^Faculty of Veterinary Medicine, Okayama University of Science, Imabari, Japan; ^2^Graduate School of Integrated Sciences for Life, Hiroshima University, Higashi-Hiroshima, Japan

**Keywords:** colchicine, cow, goat, milk, bacteria, phagocytosis, mastitis

## Abstract

Despite the occurrence of mastitis, no bacteria were detected in any of the milk samples after culture. This is partially because the neutrophils present in milk phagocytose bacteria during milk preservation. In this study, we investigated whether colchicine inhibited the decrease in viable bacteria in milk by suppressing phagocytosis during preservation. The number of viable bacteria decreased when cow milk was preserved for 5 h. However, the addition of 0.1 and 1% colchicine significantly increased the number of viable bacteria (*p* < 0.05). The percentage of culture-negative cow’s milk increased more than two-fold after 5 h compared to that at 0 h of preservation, however this percentage was significantly reduced by the addition of colchicine (*p* < 0.05). When goat milk with mastitis was incubated with bacteria (*Escherichia coli*, *Klebsiella pneumoniae*, and *Staphylococcus aureus*), the percentage of phagocytosed neutrophils decreased significantly with the addition of colchicine (*p* < 0.05). These results indicate that colchicine suppressed the decrease in the number of viable bacteria by preventing neutrophil phagocytosis during milk preservation. These findings may help in the identification of mastitis-causing bacteria and the selection of antibiotics for the treatment of mastitis.

## 1 Introduction

Mastitis should be promptly diagnosed and treated as soon as possible. Antibiotics are the most commonly used treatment. To select appropriate antibiotics, it is necessary to identify the infecting bacterial species. However, nearly 20–50% of milk samples collected from mastitic cows show “no significant bacterial growth” in routine clinical culture assays, the exact reason for which is currently unknown (1–3). This could be attributed to infection caused by bacteria present in low numbers without a reduction in the somatic cell count (SCC) (4). Other influencing factors include the sampling procedure, treatment of milk samples, media used in the bacteriological examination, presence of pathogens below current detection thresholds, or absence of bacteria at culture initiation. Mastitis may be caused by non-bacterial microorganisms (4). It has also been reported that bacteria are not detected when milk is cultured because the cause is not viable bacteria, but bacterial components such as lipopolysaccharide (LPS) (5–7). However, despite the presence of viable bacteria in freshly collected milk, these bacteria die during storage (1). According to these studies, when milk from cows with subclinical mastitis was preserved for 5 h at room temperature, the number of bacteria decreased significantly. Furthermore, Koshiishi et al. (8) investigated the cause of this decrease in bacterial counts, somatic cells (leukocytes), and antimicrobial substances in milk whey, and found that leukocytes were the main factor in decreasing bacteria in milk during preservation. Therefore, if the function of these leukocytes (phagocytosis) is inhibited, the decrease in the number of viable bacteria during storage in milk with mastitis can be suppressed.

For phagocytosis, leukocytes must alter the cytoskeleton and change the shape of the cell to surround the pathogens. The cytoskeleton is composed of proteins, such as tubulin. Colchicine binds to tubulin, thereby blocking the assembly and polymerization of microtubules, resulting in the suppression of leukocyte chemotaxis, migration, adhesion, and phagocytosis (9).

Therefore, it is thought that colchicine reduces leukocyte phagocytosis and keeps viable bacteria alive in milk. However, this has not yet been investigated.

Therefore, in this study, we examined whether the addition of colchicine to mastitis milk suppressed the decrease in the number of viable bacteria. The results of this study will help identify the causative organisms of mastitis and may provide useful information for its treatment.

## 2 Materials and methods

### 2.1 Animals

In Experiment 1, 55 dairy cows (age: 4- to 11-year-old, parity: 2–7) with subclinical mastitis were kept on a private dairy farm in Ehime Prefecture. Subclinical mastitis was diagnosed with a positive California mastitis test (CMT) and SCC of 300,000 cell/ml or higher, however with no clinical symptoms. Nine healthy Tokara goats (age: 1–9 years, parity: 1–7) were used in Experiment 2. The experiment was approved by the Hiroshima University Laboratory Animal Committee and conducted in accordance with the relevant regulations (E19-3-4, C19-4-4).

### 2.2 Experimental design

#### 2.2.1 Experiment 1

Quarter milk was collected from mastitic udders into tubes containing colchicine (Sigma-Aldrich, St. Louis, MO, United States). Colchicine was dissolved in ethanol at a concentration of 0.1 and 1 mg/mL. Ten microliters of each sample was added to a plastic tube (10 mL) and allowed to dry for approximately 1 h. When 10 mL of the collected milk was added to this tube, the final concentration of colchicine was found to be 0.1 and 1 μg/mL, respectively. After preserving the milk at room temperature for 5 h, 100 μL of milk was inoculated on sheep blood agar medium and incubated for 24 h to count the colonies. Tubes without colchicine were also prepared and the milk in those tubes were left for 0 and 5 h and then inoculated onto the agar medium, as described above. Bacteria were identified as previously described (1).

#### 2.2.2 Experiment 2

Goat milk was centrifuged (220×*g*, 4°C, 5 min) and the precipitate (SCC) was washed once with PBS. SCC was adjusted to 10^6^ cells/ml with PBS, and colchicine was added at 0, 0.1, and 1 μg/mL followed by the addition of formalin-inactivated *Escherichia coli*, *Klebsiella pneumoniae*, and *Staphylococcus aureus* (4 × 10^6^ cell/mL). After incubation at 37°C for 30 min, SCC was smeared onto glass slides for Giemsa staining, according to the conventional method. Neutrophils with or without bacterial phagocytosis were counted under a microscope. The ratio of the phagocytosed neutrophils to the total number of neutrophils was calculated.

### 2.3 Statistical analysis

**Figure 1:**
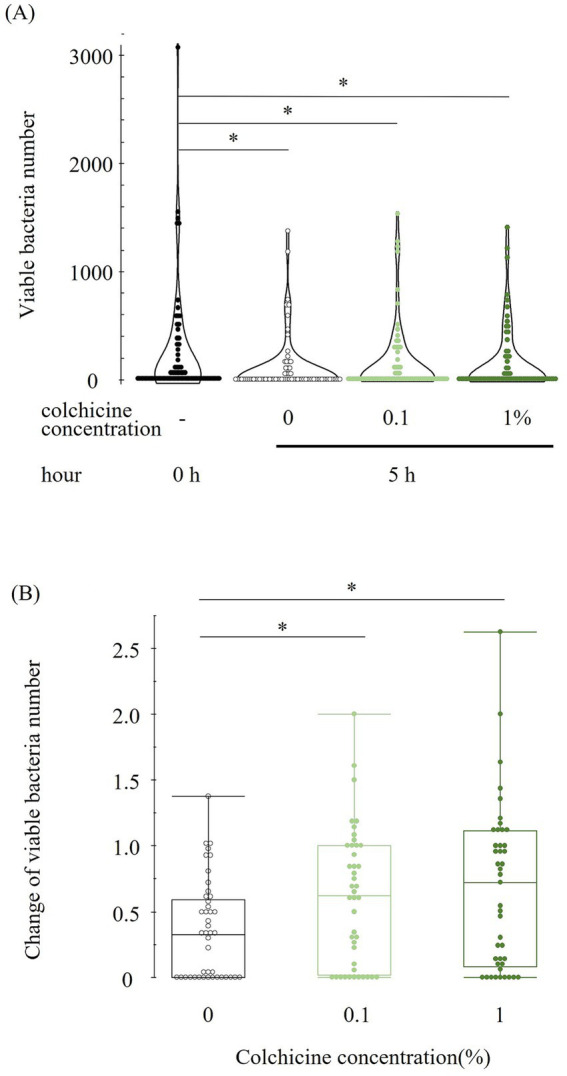
Viable bacterial number at different concentration of colchicine at 0 and 5 h (A). Change in viable bacterial number in each concentration of colchicine from 0 to 5 h for each sample (B). Values below or above 1 indicate a decrease or increase in viable bacterial counts at 5 h compared to 0 h. * shows significant difference between groups (*p* < 0.05).

**Figure 2:**
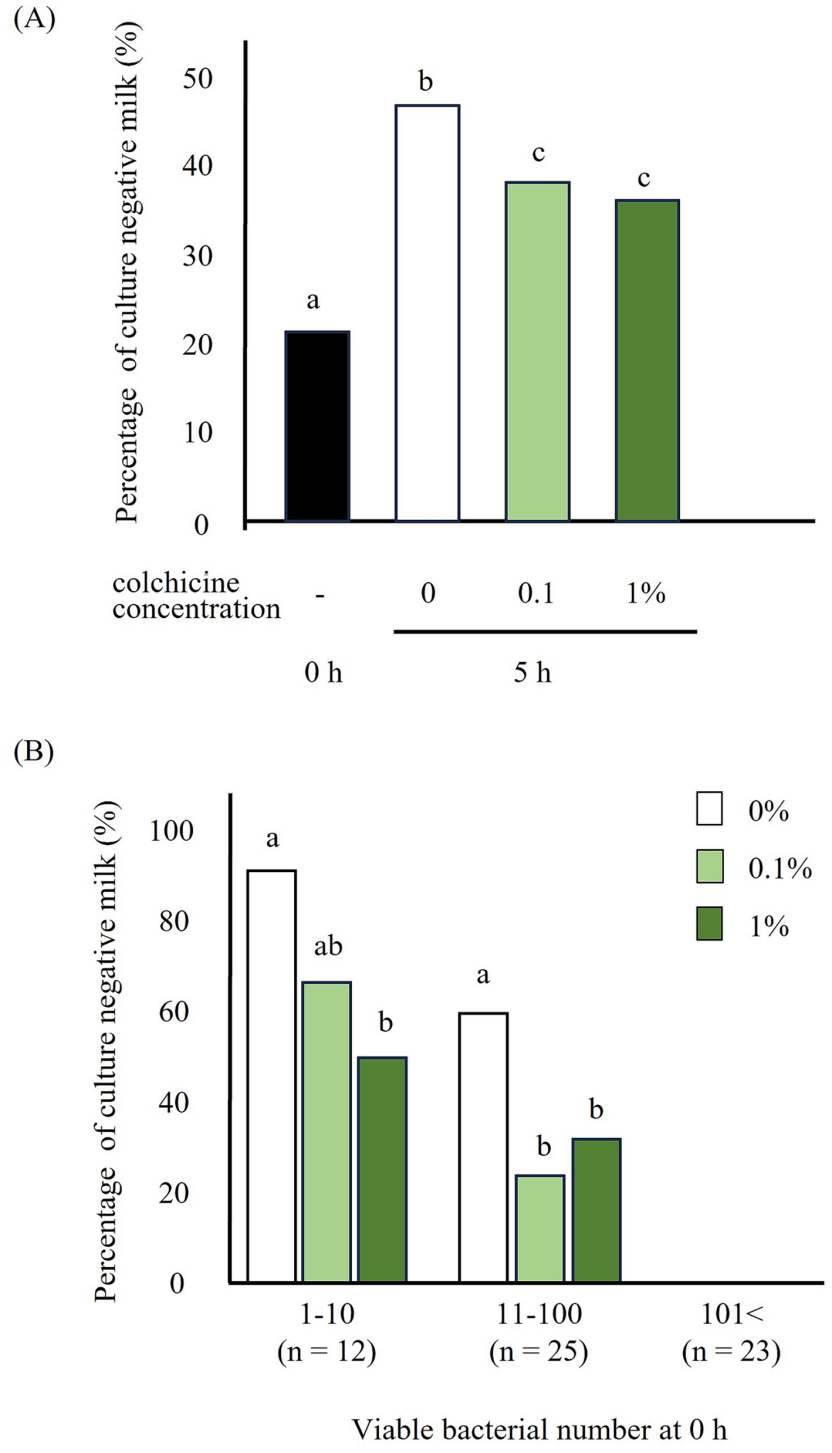
Percentage of culture negative milk preserved with and without colchicine for 5 h (A) and milk classified based on the bacterial number (1–10, 11–100, 101<) at 0 h (B). a, b, c: significant difference between different letters in different viable bacterial group.

A one-way ANOVA was performed using JMP®Pro16 (SAS Institute) to compare the values between the colchicine groups (Figures 1, 4), followed by multiple comparisons between groups using the Wilcoxon and Steel-Dwass tests. The percentage data in Figure 2 were analyzed using the chi-square test to compare the different colchicine groups. Differences were considered statistically significant at *p* < 0.05.

## 3 Results

The bacterial counts immediately after milk collection (0 h) and after 5 h of preservation with and without colchicine are shown in Figure 1A. Compared to immediately after milk collection (0 h), the viable bacterial counts were lower in all 5 h groups regardless of the addition of colchicine. However, there were no significant differences between any of the 5 h groups. Figure 1B shows the change in viable bacteria from 0 to 5 h for each sample, with values below or above 1 indicating a decrease or increase in viable bacterial counts, respectively, at 5 h compared to 0 h. The ratio of change in viable bacterial counts was significantly higher in both colchicine groups than in the 0% group.

The percentage of milk with negative cultures increased significantly after 5 h of preservation without colchicine compared to that immediately after milk collection (Figure 2A). However, addition of colchicine led to a significant decrease in this percentage, regardless of the colchicine concentration. When milk was classified by the number of bacteria before preservation, the percentage of culture-negative milk significantly decreased in the 1% colchicine group for bacterial counts of 1–10 and in both colchicine concentration groups for bacterial counts of 11–100 (Figure 2B).

**Figure 3:**
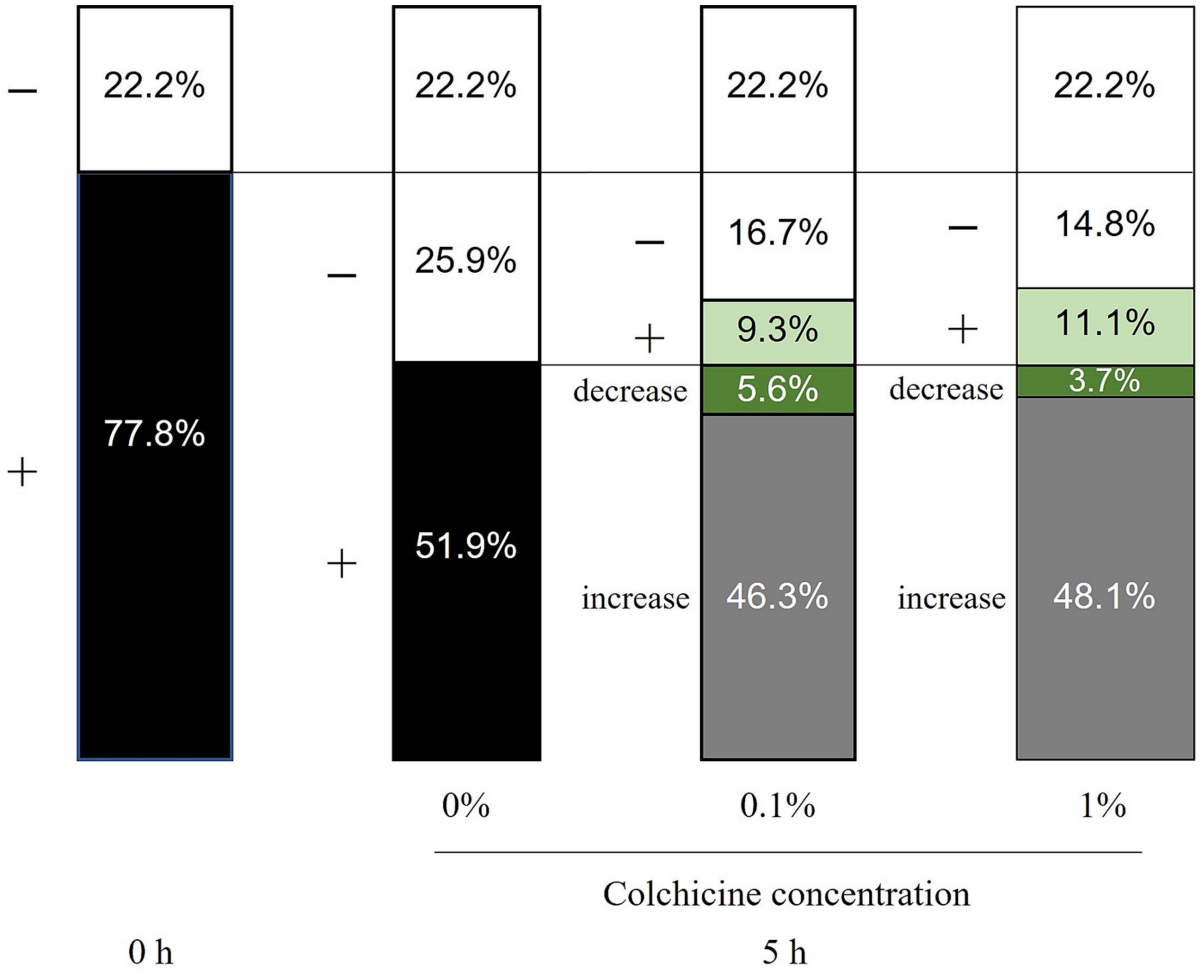
Changes in bacterial count with and without colchicine preserved for 5 h. −: milk with culture negative, +: milk with culture positive, Decrease, increase: compared with those preserved without colchicine for 5 h.

Changes in the number of viable bacteria after 5 h of incubation with colchicine are shown in Figure 3. Of the milk samples in which viable bacteria were detected before incubation, 25.9% were culture-negative after 5 h. Of these, 9.3 and 11.1% were viable after treatment with 0.1 and 1% colchicine, respectively. Addition of colchicine increased the number of viable bacteria (46.3% at 0.1 and 48.1% at 1% colchicine) in milk and viable bacteria were detected after 5 h of incubation.

When preserved for 5 h, both gram-negative and gram-positive bacteria were observed in the culture-negative milk without colchicine, however became positive when 0.1% colchicine was added (9.3%). In the milk that was positive without colchicine at 0% but increased at 0.1% colchicine (46.3%, data not shown). However, only gram-negative bacteria were observed in the milk that remained negative after addition of 0.1% colchicine, where the bacterial number decreased with the addition of colchicine (data not shown).

**Figure 4:**
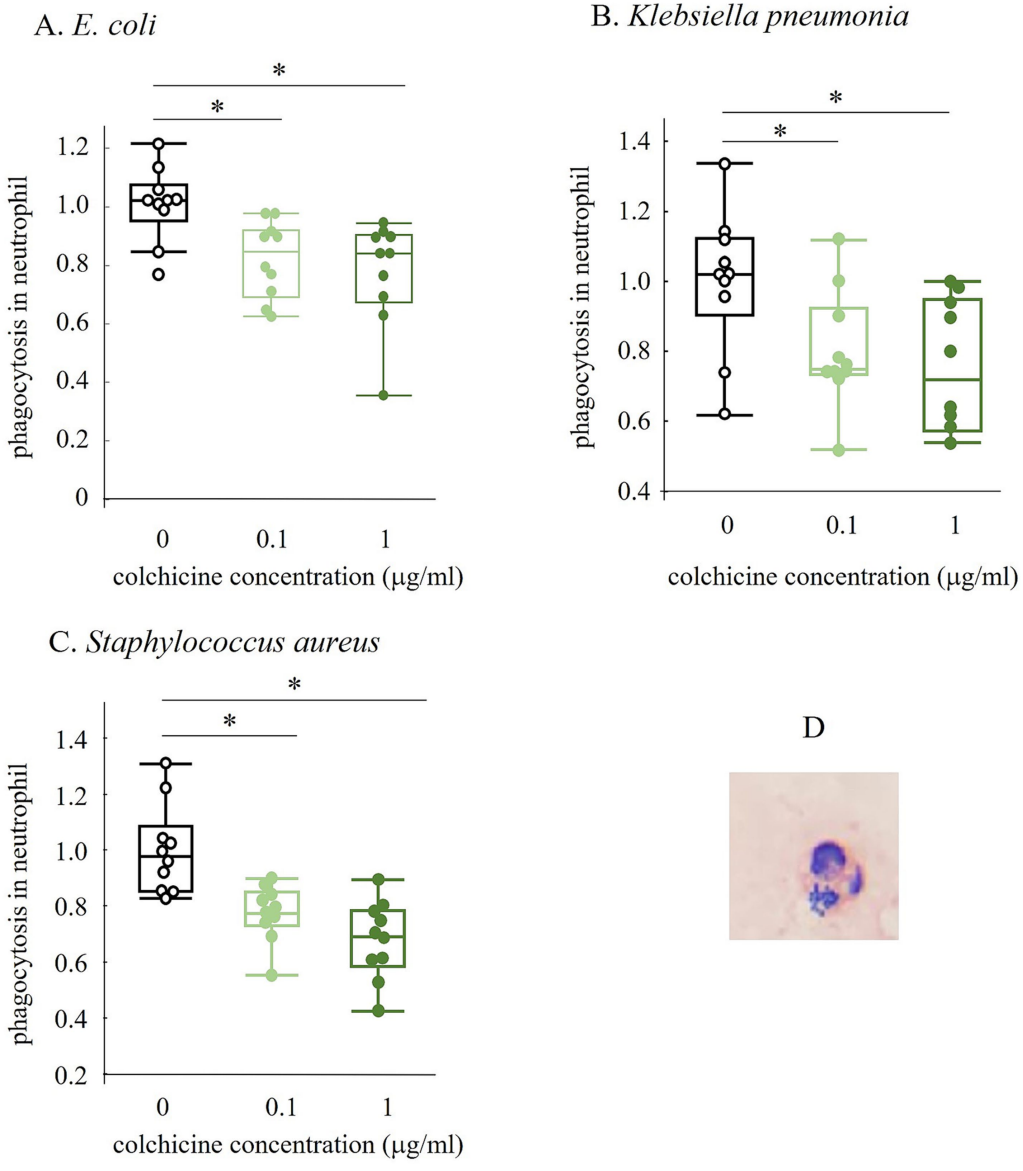
Changes in phagocytosed neutrophil number with and without colchicine preserved for 5 h (A–C). The image in D shows neutrophil that have phagocytosed *Staphylococcus aureus* (SA). All values were ratios based on the 0 micro g/mL of colchicine group. * shows significant difference between groups (*p* < 0.05).

We investigated whether the effect of colchicine in improving bacterial count could be attributed to the inhibition of leukocyte phagocytosis (Figure 4). Addition of colchicine, regardless of its concentration, reduced the percentage of leukocytes that phagocytosed *E. coli, K. pneumoniae*, and *Staphylococcus aureus*.

## 4 Discussion

When milk was collected and preserved for 5 h, the number of bacteria reduced compared to that before preservation. Conversely, the percentage of culture-negative milk increased significantly at 5 h compared with that before preservation. Thus, when milk is preserved, the number of bacteria decreases drastically, and the percentage of culture-negative milk increases. Hisaeda et al. (1) counted the number of bacteria after preserving mastitis milk for 0, 0.5, 1, 2, 3, 4, and 5 h and observed that the number of bacteria was reduced by half at 30 min and then further decreased. Culture-negative milk makes it difficult to identify bacteria. Therefore, colchicine was used in this experiment to suppress the decrease in viable bacteria. Results showed that the number of viable bacteria increased however the percentage of culture-negative milk decreased in the presence of both 0.1 and 1% colchicine. Therefore, colchicine suppressed the decrease in the number of bacteria during milk preservation.

Colchicine is expected to inhibit phagocytosis by neutrophils and macrophages because it binds to tubulins that block the assembly and polymerization of microtubules, which are key components of the cytoskeleton (9). Therefore, we investigated whether colchicine inhibits neutrophil phagocytosis. The number of phagocytosed neutrophils significantly decreased with the addition of colchicine. This suggests that colchicine inhibits phagocytosis by neutrophils, thereby suppressing the decrease in the number of viable bacteria.

Addition of colchicine significantly reduced the culture-negative rate at viable counts of 1–10 and 11–100 (Figure 2B). Therefore, colchicine appears to be effective against a wide range of viable bacteria. However, when the number of viable bacteria reached 101 or more, the percentage of culture-negative milk samples was 0%, regardless of the addition of colchicine. Therefore, when the number of viable bacteria was 101 or higher, there was almost no culture-negative milk due to the high number of bacteria, and colchicine was not necessary. However, the percentage of culture-negative milk in the 1–10 group was higher than that in the 11–100 group. Therefore, with respect to low viable bacterial counts, all bacteria tended to be phagocytosed by neutrophils.

Figure 3 shows that 48.1% of milk was culture-negative after 5 h of preservation without colchicine. However, when colchicine was added, viable bacteria were detected in 9.3 and 11.1% of milk. Moreover, addition of colchicine increased the number of viable bacteria in most of the milk samples (51.9%), in which viable bacteria were detected after preservation for 5 h without colchicine. This indicated that bacteria were more likely to be detected with the addition of colchicine.

0.1% colchicine was effective against both gram-positive and gram-negative bacteria, but not against some gram-positive bacteria. This indicated that some gram-positive bacteria were not affected by leukocytes affected under the presence of colchicine.

Decrease in the number of bacteria during milk storage is mainly due to neutrophils, but can also be caused by antimicrobial substances (8). Antimicrobial substances in milk include lingual antimicrobial peptides (LAP), S100S7, cathelicidin, and lactoferrin, and their antimicrobial properties have been reported (10–15). If the activity of these antimicrobial substances is inhibited, the number of viable bacteria in milk can be maintained when they are used in combination with colchicine.

Even if the collected milk is placed in a refrigerator, it takes about 30 min or more for the temperature to drop. When mastitic milk was preserved for only 30 min at room temperature, the number of bacteria decreased significantly (1). Therefore, the present experiment is significant because it helps to suppress bacterial phagocytosis during this 30-min period.

In conclusion, the use of colchicine suppressed the reduction in bacterial counts in milk, making it easier to detect bacteria and reducing the culture-negative rate. This is the first study to identify a method for maintaining viable bacteria in milk that can contribute to the selection of antibiotics for the treatment of mastitis. Not all causes of culture-negative milk are due to bacterial death during milk preservation, but it is expected that using this method may slightly reduce the culture-negative milk.

## Data Availability

The raw data supporting the conclusions of this article will be made available by the authors, without undue reservation.
